# Exploring the impact of a daily sport uniform policy in primary schools: a qualitative study with students and teachers

**DOI:** 10.3389/fspor.2025.1633045

**Published:** 2025-07-14

**Authors:** Carly Gardner, Cassandra Lane, Alix Hall, Nicole McCarthy, Emma Pollock, Rachel Sutherland, Luke Wolfenden, Nicole Nathan

**Affiliations:** ^1^ School of Medicine and Public Health, Faculty of Health and Medicine, University of Newcastle, Newcastle, NSW, Australia; ^2^ The National Centre of Implementation Science (NCOIS), The University of Newcastle, Newcastle, NSW, Australia; ^3^ Hunter Medical Research Institute, New Lambton Heights, NSW, Australia; ^4^ Hunter New England Area Health Service, Hunter New England Population Health, Wallsend, NSW, Australia

**Keywords:** school uniform policy, physical activity, physical education, student well-being, student equity, school teachers

## Abstract

**Background:**

Identifying effective, scalable interventions that promote physical activity in children, is a public health priority. Implementing daily sport uniform wear (activity enabling uniforms) in schools represents one such option. This qualitative study explored the perspectives of students and teachers at primary schools (elementary schools) where a daily sport uniform policy had been implemented.

**Methods:**

We used a pragmatic, exploratory qualitative study design with data collected from participants in the intervention arm of a randomised controlled trial. One-on-one interviews with teachers were conducted following a semi-structured guide and student focus groups used an activity-based structure. Thirteen students and two teachers from two schools participated in data collection between August–September 2023. The two data sets (students and teachers) were analysed inductively using Framework Analysis identifying three overarching themes.

**Results:**

The first two themes were synthesised from both data sets while teacher's perspectives primarily contributed to the final theme. First, daily sport uniform wear was highly accepted, due to their visual appeal, simplicity and cost-savings relative to formal uniforms. Second, daily sport uniform wear positively impacted students by enabling physical activity, enhancing wellbeing, providing comfort, and promoting equity. Third, formal uniforms were perceived as the norm, associated with tradition and societal expectation.

**Conclusion:**

This study deepens our understanding of the potential health and wellbeing benefits a daily sport uniform policy may have and highlights barriers such as traditions persist in school settings. Policy sustainment may require considered implementation support and consulting students in uniform designs and options.

## Introduction

Supporting Physical Activity (PA) behaviours in children can facilitate health across the life course, serving as a protective factor against non-communicable diseases such as cardiovascular disease, diabetes Type II, and breast and colon cancer (1). PA also has psychosocial health benefits including reduced depression and anxiety symptoms and increased feelings of confidence (2–4). Globally, many children and adults are not participating in sufficient levels of PA to attain the associated health benefits. The World Health Organization (WHO) estimates only 19% of children meet the recommended 60 min of Moderate to Vigorous PA (MVPA) per day (5). Further, there is a notable gender disparity in PA participation, with females consistently accumulating less PA than their male counterparts (5, 6). For example, the WHO’s 2022 Global Status report on PA showed that for children aged 11–17 years, a greater proportion of girls were inactive compared to boys (85% vs. 77.6%) (5). This gender disparity in PA is evident in the school setting as well, where research consistently demonstrates that girls are less active than boys at school (7–9).

Schools represent a valuable setting to implement programs aimed at increasing PA of children. Consequently, significant investment has been made in various school PA programs (10, 11). Consistent with WHO guidelines, recent efforts have focused on creating daily opportunities for children to be active, such as integrating quality PA into lessons for example energisers and promoting active recess and lunch breaks (12–14). While these initiatives are effective, increased gains may be achieved, especially for girls, if a persistent barrier is addressed: the restrictions imposed by formal school uniforms (7). These uniforms can hinder movement and reduce students’ ability to engage fully in PA, limiting the impact that such initiatives can have on population PA levels.

Formal school uniform policies are common worldwide, including in countries such as Japan, South Korea, Mexico, Chile, Ireland, The United Kingdom (England, Wales, Scotland and Northern Ireland), Australia, New Zealand, parts of Canada (15–17). These uniform policies are typically based on binary options consisting of blouses and skirts, tunics, or dresses for girls (female uniform), and trousers, button-up shirts, and neck ties for boys (male uniform), both of which are often worn with formal leather shoes. Evidence suggests that such formal uniforms can impede children’s PA (6, 16, 18), with the effects potentially more pronounced for those wearing a female uniform (8). Additionally, some research indicates school uniforms may also impact the psycho-social outcomes of students such as pressure from peers, concerns about unflattering uniforms (6) and cost pressures (17), with both positive and negative effects found, depending on uniform design and the social-environmental context. Importantly, some studies indicate negative impacts may disproportionately affect girls, children of diverse genders, and those from minority groups or low socioeconomic backgrounds (16).

Some schools may have designated days (e.g., Sports Days) or times, (e.g., during Physical Education classes) when students are allowed to wear an activity-enabling uniform, often referred to as a “sports uniforms” or “sports kits.” These typically consist of polo shirts, shorts or sport skirts, and running shoes (17). Research suggests that students are more active when wearing sport uniforms compared with their formal uniform (6, 8). For example, a 2023 cross-sectional study in 374 Chilean high schools with 8030 students found sport uniforms were associated with higher cardiorespiratory fitness among high school students compared to formal uniforms (18). In Australia, a 2012 study conducted in Western Australia, which measured the PA of 64 children over a four-week period, found uniform type had more of an impact on girls PA than boys reporting mean daily steps for girls were significantly lower on days when they wore a formal uniform (933.3 steps, SD 271.8) compared to days when they wore sports uniforms (1,134.1 steps, SD 271.9) (*p* = 0.006) (8). Similarly, a randomised controlled trial (RCT) including 42 schools in New South Wales (NSW), tested the effects of students wearing a sport uniform all day or a traditional uniform all day, on accelerometers measured activity levels. Results from 1,847 primary school students found a statistically significant increase in break time minutes of light PA for girls in the intervention compared to control group (0.62, 95% CI: 0.15, 1.10; *p* = 0.012) (no effect found for boys) and a reduction in minutes of sedentary activity for all intervention students compared to control students (−0.81; 95% CI: −1.45, −0.17; *p* = 0.014) (19).

Given the potential health and wellbeing benefits of sport uniforms, changing school uniform policies to allow daily sports uniform wear represents a simple and cost-effective health promotion strategy. Existing evidence primarily focuses on the impacts of formal uniforms and the PA benefits of sports uniforms (6, 16, 20), with limited exploration of the broader health and wellbeing impacts of schools’ adoption of a daily sport uniform policy. In a 2021–22 primary school-based trial in NSW Australia, intervention schools with formal uniform policies were supported to implement a policy allowing students in years five and six to wear a sport uniform daily (19). This study aims to explore the perspectives and experiences of students and teachers at intervention schools where this policy was implemented.

## Materials and methods

### Context

This qualitative study was conducted alongside a nine-month cluster randomised controlled trial (cRCT) evaluating the impact of a school uniform policy intervention in New South Wales, Australia (19). The trial aimed to assess whether allowing students to wear their sport uniform every day would improve cardiorespiratory fitness and physical activity in children in years 4–6 (aged 9–12). A total of 24 primary schools were randomised to either an intervention or control group. Intervention schools received support to implement a policy change permitting daily wear of the school’s existing sport uniform. Control schools maintained their usual uniform policies. Primary outcomes included objectively measured cardiorespiratory fitness (i.e., validated 20 m multistage fitness test) and physical activity (i.e., mean daily step count measured via accelerometer). This qualitative study was embedded within the cRCT to explore students’ experiences of the policy change and to inform understanding of factors influencing implementation and sustainability.

### Study design

We conducted an exploratory qualitative study with a pragmatic methodological approach (21). Data were collected via semi-structured one-on-one interviews with teachers and activity-based focus groups with students at schools that had participated in the daily sport uniform policy trial (intervention schools). The research methods were developed and employed in accordance with best-practice guidelines for qualitative research in public health and adhered to the Standards for Reporting Qualitative Research (22, 23). Ethics approvals were received from the local health district (2020/ETH02602), the University of Newcastle, Human Research Ethics Committee (H-2021-0013), and the relevant regional school offices.

### Reflexivity statement

We, the authors, are a team of predominantly women implementation scientists embedded within a health service organisation (24). Our team has extensive experience in public health program delivery and evaluation in the school setting, with several members being trained school teachers. We have vocational and personal interests in child health, creating health enhancing school environments, physical activity, and intersectional gender equity. While we advocate for practices that promote PA in schools, we are dedicated to understanding the impacts—both positive and negative—of health interventions through the experiences and insights of the school community.

### Note on gender/sex

Although inter-connected, we recognise the important distinction between sex (a biological construct) and gender (socio-cultural construct) (25). In this study we use language that aligns with the binary uniform policies of the participating schools, where students wear either a female or male uniform. To respect student identity, we did not ask students to identify their gender but instead to indicate which of the two uniform options they wore. The terms “female/girl” and “male/boy” in this study reflect uniform categories rather than assumptions about gender identity.

### Sampling and recruitment

We purposively sampled primary schools from the trial, inviting those that had implemented a daily sport uniform policy to participate in the study. We excluded intervention schools that had not fully implemented the policy or those where project staff identified that the school was experiencing post-covid challenges, such as high burden on staff. We aimed to include both “urban” and “regional” schools (classified by Australian Bureau of Statistics: Australian Statistical Geographical Standard system (26). We first obtained consent from principals. Using our existing connections, we emailed them a study information statement and consent form. Upon receiving their consent, principals were asked to nominate a teacher contact, who then served as the liaison between the research team and eligible staff and students at their school. We then sought individual consent from participants within consenting schools for the data collection activities detailed below. In cases where we did not receive a response, we made one additional attempt to contact the schools.

### Data collection

#### Interviews of teachers

Our data collection approach for teachers followed methods that we have successfully used in our previous school-based research (27). We developed a list of eligible teachers (those who taught a class that had participated in the trial) from consenting schools and invited them to participate in a one-on-one interview, either in-person or via Zoom, based on their preference. The lead researcher (CG) conducted the interviews following a semi-structured interview guide that had been co-developed and piloted by the research team. Participants received a gift card ($30 AUD) as compensation for their time.

#### Student focus groups

We used an external distributor model to recruit students, collaborating with schools to identify eligible students who had been exposed to the daily sport uniform policy [i.e., current Year Six students (aged between 11 and 13 years) at consenting schools who were enrolled during 2022]. The nominated liaison in the school selected the students to participate based on the eligibility provided. Schools shared study information and consent forms directly with eligible students using existing school communication channels. Parents gave active, written consent for their child to participate, and student consent was obtained both in writing prior and verbally at the start of the focus group. Participants were those who were eligible, had consent and were present at the school on that day of data collection.

We coordinated with consenting schools to schedule each focus group at a convenient date and time. To minimise logistical challenges for the school and students’ families, focus groups were held during school hours and took place on school grounds, in a familiar and quiet environment such as the school hall or an empty classroom. This arrangement aimed to increase comfort and encourage participation in the discussions (28). Two trained members of the research team (CG) and (EP, a trained teacher) facilitated focus groups with a school teacher present to provide additional support.

Each focus group lasted approximately 20 min. The facilitators followed a semi-structured guide consisting of three sections, described in Table 1. The guide was developed following a review of the literature on qualitative research with children (6, 20, 28). It was designed to engage all students and support their sustained interest by employing multiple methods including verbal, written, and movement-based response options (29). Our aim was to ensure that all students had an opportunity and felt comfortable to share their experiences and thoughts relating to their sport uniforms and wearing them daily. The questions were tested for readability and understandability for a child at a reading age of 10 (30) and piloted with primary school-aged children of the research team (28).

**Table 1:** Summary of student focus group procedures and questions.

Section	Description of activity	Question
Section 1:	Where do you stand: Students are asked to stand and move to either end of the room depending on their response to the question. One facilitator conducts brief discussions with each group.	Who likes wearing their sport uniform every day?
	EQUIPMENT: N/A	
Section 2:	Post it note: Students are asked to write responses to question on sticky notes and post to a large paper under categories: “*at school,” “at home,” “before and after school activities*,” *“other.”* “Post box” is available for confidential submission. Facilitators provide prompts or clarify as needed.	What has wearing your sport uniform everyday meant for you? Has it changed anything for you?
	EQUIPMENT:	
	- Large sheets of paper with domains written along top - Pencil and small sticky note stack for each participant - “Post box” consisting of a small, closed box with hole cut out (for depositing notes confidentially)	
	Verbal discussion	Does anyone have anything they want to add or discuss about the post-its?
	Verbal discussion	Do you think the school should continue with wearing sports uniforms everyday? Why/why not?
Section 3:	Verbal discussion: Students are shown photographs of the formal female and male uniforms. Students who wear the male uniform leave the room with one facilitator (EP) and one facilitator remains to facilitate discussion with the students who wear a female uniform (CG).	Is wearing your sport uniform every day different when you wear this formal uniform to that one *[pointed to each uniform type in the photograph]*? If so, can you describe how, if not why not?
	EQUIPTMENT:	
	- Photograph of formal school uniforms	

The facilitators began each session by emphasising the voluntary nature of the focus group and the need for confidentiality. Throughout the session, facilitators reiterated that there were no right or wrong answers, holding space for diverse experiences. To address power imbalances between adult facilitators and child participants, facilitators used their first names and sat in a circle alongside the students (28).

To engage a range of learning and communicate needs, the interactive focus groups were designed in three sections. The first section (Section 1) began with an ice breaker activity where students moved to different sides of the room based on their response to a question, as shown in Table 1. This was designed to engage students and allow opportunity for divergent responses (29). Section two, was structure around quiet reflection and written responses, aiming to give “voice” to potentially quieter members of the group who may be less inclined to respond verbally (29). Participants wrote their answers on sticky notes, one thought per note, and no limit on the number they could contribute. Students then placed their notes on large sheets of paper under the respective domain: “*at school*,” “*at home*,” “*before and after school activities*,” or “*other*.” Alternatively, students were able to submit their notes anonymously in a box. The domain categories also functioned as prompts for students to consider the range of impacts of the daily sport uniform policy. Students continued to create responses until they had no more ideas to add. Afterward, students regrouped to provide additional verbal feedback and respond to a follow-up question.

The final section (Section 3) asked students which formal uniform they wore and those who wore the “male” uniform left the room, allowing remaining participants to share additional impacts that were specific to their experience wearing the female uniform (i.e., formal tunic dresses). As previous research suggests formal uniforms create additional barriers for girls at school (6, 20) this section gave them the opportunity to voice any impacts they may have felt uncomfortable sharing in the larger group. It also facilitated straightforward consideration of inter-gender differences in the student experience of the daily sport uniform policy.

The focus group concluded with a shared plate of fruit and light lunch food, in gratitude for the students’ time accounting for missing a standard break time.

### Data analysis

All data were audio-recorded using hand-held devices and then transcribed by a professional transcription company (Outscribe) (31). Transcripts were cross-checked and de-identified to maintain participant confidentiality, and then uploaded into QSR NVivo (32) for analysis. Two members of the research team, including the study lead (CG) and a proficient qualitative researcher (CL), conducted an inductive analysis using Framework Analysis (33). First, the analysts co-developed an initial codebook based on the research objectives. They piloted the process together by co-coding one transcript and making changes to the codebook to ensure it could be used efficiently. Both analysts then independently used the final codebook to code the remaining transcripts. After coding all transcripts, they met to discuss and collaboratively develop an initial list of themes. One analyst (CG) populated an outcomes table with themes and supporting quotes which was presented to the research team for collaborative exploration and refinement of the themes, and to assign final names and descriptions.

## Results

Of the four eligible schools invited to participate in this study, two consented and proceeded to coordinate data collection activities. Reasons for not consenting included competing priorities for one school, and the departure of key contacts at the other. Two of the three teachers exposed to the daily sport uniform policy in the participating schools participated in interviews. Two student focus groups were conducted, one at each school. The total sample comprised of 15 participants: two teachers (both identified as male) and 13 students (seven male and six female). Data were collected between August and September 2023.

Overall, participants strongly approved of the daily sport uniform policy, expressing support based on their personal views and positive experiences while the policy was implemented in their school. Three distinct themes encompassing nine sub-themes were drawn from the data. These are described below and summarised with additional quotes in Table 2.

**Table 2:** Summarised themes and subthemes with sample quotes.

Themes & Sub-themes	Sample Quotes: *verbatim quote* (theme(s) relates to)
1. Daily sport uniform wear was highly accepted (and often endorsed) by the school community. A. Visual appeal B. Simplicity C. Cost-savings	**Male student 9:** *It [sport uniform] looks better I think* (Sub-theme 1.A).
	**Female student 7:** *Yes [I like the sport uniform], because it’s more free and people like it better* (Sub-theme 1.B).
	**Teacher 1:** *As a parent, I would much rather it [daily sport uniform policy], because the [sport] uniform is so much easier* (Sub-theme 1.B).
	**Student written comment (FG2):** *It is more easy to play at* home (Sub-theme 1.B)
	**Student written comment (FG2):** *It is eser [easier] if you go to a friends hous [house]* (Sub-theme 1.B)
	**Female student 6:** *[the formal uniform is] So expensive. If the other sport uniform, sport uniform, winter uniform, summer uniform you probably need two of them in case they get dirty. So it’s like way more expensive [having the formal uniform]* (Sub-theme 1.C)
	**Student written comment (FG1):** *less money because you only need one or two uniforms* (Sub-theme 1.C)
2. Daily sport uniform wear positively impacted on students—especially for girls A. Enables physical activity B. Enhances student wellbeing C. Provides comfort/practicality D. Promotes equity	**Female student 4:** *Yes [I like the sport uniform] because it’s way easier to put on. It’s more stretchy, flexible and I feel really free when I have it on doing cartwheels and handstands and everything at school* (sub-theme 2.A, 2.B, 2.C & 2.D).
	**Female student 6:** *I feel more wanted to do sport when I’m in my sport uniform* (sub-theme 2.A). *But when I’m in my winter and summer uniform [formal uniforms] it gives me another reason why to not be as active. And it was just so much better last year [when the daily sport uniform policy was implemented]. It just felt so much more free and better in general* (sub-theme 2.A, 2.B & 2.C).
	**Student written comment (FG1):** *I overall leaned towards the sport uniform because I could play more* (sub-theme 2.C).
	**Male student 5:** *I feel way more like relaxed and happy when I put it on because [I] just got a better mindset* (sub-theme 2.B)
	**Student written comment (FG1):** *I feel more free whilst wearing the sport uniform)* (sub-theme 2.B/C).
	**Student written comment (FG1):** *I can focus better in class [in the sport uniform]* (sub-theme 2.B).
	**Student written comment (FG1):** *my mood changes because I feel more comfortable and relaxed [in the sport uniform]* (sub-theme 2.B & 2.C).
	**Teacher 2**: *Definitely morale has been very low this year, so trying to build morale, and that’s why I’ve been taking them out for more sports sort of thing. I feel having sports uniforms every day would be definitely beneficial, especially for example, one of our rewards if we talk respectfully to each other was to do a class Olympics. So therefore if we had sports uniform every day I could do a little bit of sport every single day, just maybe 15, half an hour, that could boost morale* *[sport uniform worn one day per week at time of comment].* (sub-theme 2.A, 2.B).
	**Teacher 1:** *From a relaxed point of view in a classroom it was very comfortable, in my point of view, very, very beneficial.* (sub-theme 2.C).
	**Female student 7:** *With the girls, if you choose to wear the summer dress you can’t do as much stuff as if you’re just wearing the sport uniform every day. Like some people might be uncomfortable running in the dress* (sub-theme 2.A, 2.C & 2.D).
	**Female student 10**: *I like it as an option. See what I’m wearing [sport uniform], I actually kind of like it because I’m not suitable in a dress. I’m not a dressy kind of girl. I’m a jeans and a t-shirt or shorts and a basic shirt* (sub-theme 2.D).
	**Female student 11**: *It was very hard to catch stuff, get stuff in the dress. You have to pick sizes that actually go down past your knee so you can keep your arms up so it doesn’t go higher than yourself* (sub-theme 2.D).
	**Teacher 1**: *It [the daily sport uniform policy] did help one of our students who was I suppose special needs because he didn’t usually go well with the other uniform [formal uniform], he’d eat it and different things like that. So from a sensory point of view, it was probably a bit more beneficial for him and it made him feel a bit more comfortable* (sub-theme 2.C &2.D).
	**Teacher 2**: *When I’ve tried to run touch football things some of the kids, especially some of the girls, have said like “this is unfair,” I’m wearing the classic girl’s winter uniform and it’s restrictive’ and “why can’t we just always train on a Wednesday” that’s basically what they said, which is what our normal sports day*. (sub-theme 2.A, B, C, D).
3. Formal uniforms are considered the “norm.” A. School tradition: identity is associated with formal uniforms. B. Societal expectations: schools are expected to present themselves in a formal way to the community.	**Female student 10**: *If we got the option to choose sport or formal uniform and there was somebody from an office that’s very important to our school, I would choose to wear my formal uniform to represent my school very formally* (sub-theme 3.B).
	**Teacher 1:** *School uniform for us is obviously a button up shirt, proper pants or shorts, whatever it may be in that time of the year. So that is difficult for us because we do tend to have that whole ideal that identity of being proud of our school uniform, not that they weren’t proud in their sports uniform, but it did take away from the identity of the school that some of the community do look for.* (sub-theme 3.A & 3.B).
	**Teacher 1**: *Even to the point where we have parents who come and enrol their students here, because they say we have that uniform. And that is, I suppose, part of our value system that we do have that pride and respect, dignity in what we wear and how we present ourselves, so that is part of it. We do like those formal presence of a uniform to for our special occasions, and that sort of sets up the tone of the school as well.* (sub-theme 3.A & 3.B).
	**Interviewer:** *Do you have a sense of why it [daily sport uniform policy] didn’t continue?*
	**Teacher 2:** *I think it just went back and it was to do with just tradition, I would say.* (sub-theme 3.A).

FG1 = Focus Group 1.

FG2 = Focus Group 2.

### Theme 1: Daily sport uniform wear was highly accepted and often endorsed by the school community

This theme highlights the strong acceptance of the daily sport uniform policy by participants. Compared with formal uniforms, students preferred to wear their sport uniform for various reasons. Firstly, students preferred the visual appearance of the sport uniform (*sub-theme 1.A*), expressing dislike for the formal uniform appearance, particularly the neck ties and fitted pants or dresses. The sport uniform aligned more closely with their preferences for dressing outside of school hours. Additionally, all student participants had helped to design a school sport shirt as a memento in their final year of primary school, enhancing their preference for sport uniforms. Secondly, the sport uniforms offered simplicity (*sub-theme 1.B*), making it easy for students to get dressed in the morning and transition to afterschool activities like play or chores. Thirdly, students recognised the comparatively lower cost of sport uniforms (*sub-theme 1.C*) and saw this as a positive aspect.

The interviewed staff also expressed acceptance of the daily sport uniform policy, primarily due to its positive impact on students, as captured in Theme 2. Across all data sets, it was clear that if given a choice, most students, if not all, would choose to wear a sport uniform every day. One participating school did not sustain the sport uniform policy following the trial, much to the disdain of students: “*I’m begging you to tell our teachers to be sport uniform for the rest of the year*.” (Female Student 6).

### Theme 2. Daily sport uniform wear positively impacted on students—especially for girls

Wearing a sport uniform every day had broad positive impacts on students, distilled into four sub-themes. First, the sport uniforms facilitated engagement in physical activity, encouraging movement and exercise among students (*sub-theme 2.A)*. Participants reported this as being both practical: the design of the sport uniforms including footwear allowing for movement and emotional: wearing the sport uniform helped students feel ready for being active, explored further in the following subthemes. This sub-theme was expressed by all students and recognised by the interviewed teachers*.* Second, sport uniforms contributed to an overall sense of wellbeing, fostering positive emotional states among students (*sub-theme 2.B)*. Male Student 5 shared: “*I feel way more like relaxed and happy when I put it [sport uniform] on because … just got a better mindset*” *(sub-theme 2.B).* Statements of *s*tudent wellbeing were intertwined with the third sub-theme, that sport uniforms provided comfort and practicality (*sub-theme 2.C)*. For example, a student in Focus Group 1 wrote: “*I feel more free whilst wearing the sport uniform*” (*sub-theme 2.C*), offering ease of movement and functionality throughout the day.

Fourth, the sport uniform promoted equality among students (*sub-theme 2.D*), ensuring all students had access to appropriate attire for physical activities and fostering a sense of inclusivity within the school community. When comparing the sport uniform with a formal uniform, Female Student 4 described: “*…it’s [sport uniform] way easier to put on. It’s more stretchy, flexible and I feel really free when I have it on doing cartwheels and handstands*.” The sport uniforms also allowed girls to participate in more physical activities (e.g., class-room Physical Education on non-sports days). Moreover, the sport uniform was better suited for some students who did not feel the female formal uniform fit their persona. For example, Female Student 10 described: “*I actually kind of like it [sport uniform] because I'm not suitable in a dress. I'm not a dressy kind of girl*.”

Furthermore, the sport uniform policy had benefits for a former student with diverse needs. Teacher 1 reflected:

It [sport uniform] did help one of our students who was I suppose special needs because he didn’t usually go well with the other uniform [formal uniform], he’d eat it and different things like that. So from a sensory point of view, it was probably a bit more beneficial for him and it made him feel a bit more comfortable. (sub-theme 2.C & 2.D)

### Theme 3. Formal uniforms are considered the “norm”

Despite the positive perceptions and reported impacts of sport uniforms for students, this theme captures a key barrier for schools to adopt and sustain the daily sport uniform policy. Both interviewed teachers described their school’s discontinuation of the policy (following the trial) due to the school’s longstanding tradition of formal uniforms *(sub-theme 3.A)* and perceived societal expectations for schools to present themselves formally within the community *(sub-theme 3.B*).

Teacher 1 explained:

We have parents who come and enrol their students here, because they say we have that [formal] uniform. And that is, I suppose, part of our value system that we do have that pride and respect, dignity in what we wear and how we present ourselves, so that is part of it. We do like those formal presence of a uniform to for our special occasions, and that sort of sets up the tone of the school as well.

Several students also recognised this, for example, Female Student 10 stated: “*if we got the option to choose sport or formal uniform and there was somebody from an office that’s very important to our school, I would choose to wear my formal uniform to represent my school very formally*.”

## Discussion

This study explored the experiences and perspectives of students and teachers in two NSW primary schools following the implementation of a daily sport uniform policy. The findings highlight positive support for the policy, with both students and teachers, reporting the practical benefits of the policy, such as increased ease and efficiency for children and parents, and health-related advantages for students (Themes 1 and 2). However, implementation challenges were identified, particularly related to concerns about breaking away from societal “norms” associated with traditional school uniforms and potential negative perceptions from the broader community (Theme 3). These findings contribute to the growing body of literature on school uniforms (7, 16, 18, 34–36) by offering a nuanced understanding of the acceptability, benefits, and barriers associated with transitioning to a daily sport uniform policy.

Students overwhelmingly preferred the sport uniform, citing its visual appeal, simplicity, practicality, and affordability. These findings are consistent with a 2019 cross sectional survey of Australian primary schools, where 61.6% of 800 student respondents reported they would prefer to wear their sport uniform every day (34). Our study extends on these findings by exploring the potential reasons behind this preference, such as the perceived comfort and convenience of sport uniforms. This deeper understanding is particularly valuable for exploring the broader impact of school uniform policies on student health and wellbeing outcomes, that other authors have called for (16).

The capacity of sport uniforms to facilitate student PA and enhance student comfort have been reported previously (7, 8, 16, 35). For example, a 2022 cross-sectional study of 988 students in Chile found that 64% believed that formal uniforms negatively impacted their PA compared to sport uniforms (35). Previous qualitative work has also noted that traditional uniforms can restrict student PA (6, 20) during school time physical activity (6) or playground activity selection (20). Our findings support previous qualitative findings and broaden the potential impacts daily sport uniforms may have to other health and wellbeing outcomes. Furthermore, as our participants had experienced both a daily sport uniform and a formal uniform policy, they were able to provide rich accounts of both options. Importantly, our findings suggests that daily sport uniform wear may enhance overall student wellbeing, by fostering a sense of comfort and confidence. Other studies have explored broader wellbeing outcomes comparing traditional uniforms to sport uniforms. A cross-sectional study with 988 Chilean adolescents, reported those wearing daily sport uniforms had lower feelings of bullying and discrimination (35). While promising, these findings warrant further research to understand the role sport uniforms play in student wellbeing and to assess if factors may contribute to or mediate these effects such as the wellbeing benefits of increased PA (16). Such findings align with the health and wellbeing outcomes of many education settings and may strengthen the case for schools to adopt daily school uniform policies.

The preference for wearing sport uniforms daily was particularly strong among those students wearing female uniforms, underscoring the gendered implications of uniform policies. Consistent with previous research, traditional uniforms, such as skirts and dresses, were viewed as restrictive and uncomfortable, often limiting the wearers’ PA participation (6, 20). Given the evidence of lower PA participation among girls compared to boys (5), including in the school setting (8, 9), our study’s findings suggest promoting a daily sport uniform policy could help address the PA-gender gap. Our analysis also indicated daily sport uniform wear may enhance girls’ wellbeing beyond PA by facilitating a sense of freedom, confidence, and offering more options for the attire to better reflect their persona. Importantly these broader wellbeing themes were primarily captured when only those wearing the female uniform were in the room, indicating the importance of considering appropriate methods to generate such data.

To maximise the benefits of sport uniforms, careful consideration of uniform design is crucial. A qualitative study from the United Kingdom with 143 high school students found that poorly designed Physical Education (PE) uniforms negatively impacted girls’ comfort and participation (37). The study reported girls feeling discomfort performing activities such as cartwheels due to “baggy” pants, and concerns about tight or transparent uniforms. Other research has highlighted the importance of uniform design features such as fabric types and styles in ensuring comfort and confidence (6, 16). Co-designing uniforms with students may address these concerns and enhance their effectiveness and acceptability, as recommended by others (16, 37).

The sport uniform also demonstrated potential for enhancing equity and inclusivity. A teacher observed that the simple design of the sport uniform benefitted students with diverse needs including those with sensory needs. This finding aligns with research recommending clothing adaptations for children with sensory over-reactivity such as avoiding restrictive elements e.g., ties and buttoned cuffs and using soft fabrics (38). By reducing barriers related to cost, comfort, and accessibility, sport uniforms may contribute to broader health equity objectives for public health initiatives. Future research could further examine how uniform policies can address systemic inequities, particularly for diverse population groups.

Despite these advantages, a significant barrier to sustaining a daily sport uniform policy was the entrenched belief that formal uniforms are essential in maintaining school culture or identity and discipline. Teachers expressed concerns about community perceptions, which ultimately was cited as a key reason both schools reverted to their formal uniform policy at the end of the RCT. These findings are consistent with a 2021 systematic review of 92 articles referencing school uniforms including empirical and non-empirical publications across a range of disciplines, where the narrative synthesis reports some included articles argued traditional school uniforms can uphold school identity and values (16). However, contrasting evidence suggests that community perceptions may be more amenable to a daily sport uniform policy than previously thought (39). A cross-sectional survey of 579 teachers and 1231 parents from 62 Australian primary schools found that 63% of teachers and 73% of parents supported a daily sport uniform policy (39). The most frequently reported barrier was the perception that sport uniforms were inappropriate for formal occasions. Overcoming this perceived barrier may require strategies such as community engagement, co-designing policies, and increasing awareness of the benefits of daily sport uniform wear. Additionally, investigating community and public perceptions of school uniform policies could yield valuable insights into broader societal attitudes and support the development of strategies to change these beliefs.

## Strengths and limitations

A significant strength of this study was including participants who had experienced both a daily sport uniform and a formal uniform policy, allowing detailed comparisons to be made. However, this approach limited recruitment for the study. Ideally, we would have also included the perspectives of parents and the broader community to better understand if these attitudes align to the perceived barriers identified in Theme 3, however this was beyond our current scope. Further, while the impacts for different equity groups described in this study contributes to advancing equity in educational settings, the sample was modest in size and diversity. We did not collect demographic data on the students or schools (for example ethnic diversity or wealth) at this timepoint. The specific sample in this study needs to be considered when reviewing the findings. To balance the small sample, our methodology focused on collecting rich data and triangulating this between students and teachers to increase information power (40). Future research should consider a broader sample from a more diverse range of educational settings and student backgrounds.

Important to note, our discussion explores gender equity within the binary framework to align with the school uniform policies included in this study. Further empirical research of school uniforms through an intersectional lens would strengthen gender equity considerations, particularly with a focus on impacts for non-binary or gender diverse students who are thought to be impacted disproportionally by uniform policies (16).

A further limitation is the short time frame students were exposed to the daily sport uniforms. Long-term impacts on students’ comfort, academic performance, and overall wellbeing remain unexplored. To fully understand the sustained impact of sports uniforms, future research should include longitudinal studies that assess these outcomes over time.

## Conclusion

Achieving public health impact, requires implementing policies that balance promoting healthy behaviours and increased wellbeing with being acceptable to the target population and sensitive to the needs of different population groups. Achieving these objectives can be challenging for programs particularly in complex community settings such as schools. A daily sport uniform policy, has the potential to address these criteria while improving student comfort, increasing PA, and possibly addressing some inequities. However, sustaining such a policy will require addressing entrenched beliefs and cultural norms in school communities that favour traditional uniforms. Future studies and policy efforts should incorporate strategies to shift school and community perceptions, positioning the daily wearing of sport uniforms not as a break from tradition but as a means of supporting student health and engagement.

## Data Availability

The raw data supporting the conclusions of this article will be made available by the authors, without undue reservation.
